# Impact of Surgical Margin Status and Tumor Volume on Mortality After Robotic Radical Prostatectomy

**DOI:** 10.1016/j.euros.2024.12.004

**Published:** 2024-12-31

**Authors:** Zaki Zeidan, Joshua Tran, Yeagyeong Hwang, Linda My Huynh, Mai Xuan Nguyen, Erica Huang, Whitney Zhang, Thomas Ahlering

**Affiliations:** a Department of Urology, University of California-Irvine Medical Center, Orange, CA, USA; b University of Nebraska Medical Center, Omaha, NE, USA

**Keywords:** Prostate cancer, Percentage tumor volume, Positive surgical margins, Radical prostatectomy, Prostate cancer–specific mortality

## Abstract

**Background and objective:**

Positive surgical margins (PSMs) following radical prostatectomy (RP) have been seen as inherently unfavorable. However, a large international multi-institutional study recently revealed that unifocal PSMs (UPSMs) had no impact on prostate cancer–specific mortality (PCSM), whereas multifocal PSMs (MPSMs) did. Our aim was to assess the relative impact of PSMs versus percentage tumor volume (PTV) on PCSM.

**Methods:**

We analyzed data for 1552 patients who underwent robot-assisted RP performed by a single surgeon between 2002 and 2018 at a tertiary referral center with up to 15-yr follow-up. Patients were divided into negative surgical margin (NSM), UPSM, and MPSM groups, with PTV stratification using a cutoff of 40%. The primary outcome was stepwise multivariate regression analysis of predictors of PCSM (pT stage, pathological Gleason grade group, PTV, UPSM, and MPSM). The secondary outcome was the risk of 15-yr PCSM via Kaplan-Meier analysis.

**Key findings and limitations:**

The group with 40–100% PTV was older and presented with more advanced grade and stage. High PTV was significantly associated with greater risk of PSM, biochemical recurrence, PCSM, and overall mortality at 15 yr (*p* < 0.001). In addition to high stage and grade, MPSM predicted PCSM in multivariate analysis, but lost predictive significance when PTV was included. Limitations of the study include the retrospective nature and the single-center setting.

**Conclusions and clinical implications:**

Our study further challenges the belief that MPSMs inherently have an adverse impact on PCSM. Instead, MPSMs appear to signify more aggressive underlying disease that predominantly drives oncological outcomes. We recommend considering PTV as a more reliable predictor of PCSM. While avoidance of PSMs remains a critical surgical principle, this goal in prostate cancer needs to be weighed against urinary and sexual function outcomes.

**Patient summary:**

After surgery to remove the prostate in men with prostate cancer, samples from the edge of the prostate that are positive for tumor cells are called positive surgical margins (PSMs). Results from our study show that a PSM on its own is not necessarily an adverse factor. However, PSMs may be a sign of higher severity of prostate cancer. We found that men with a high tumor volume have a higher risk of dying from their prostate cancer.

## Take Home Message

The pathological finding of a positive surgical margin in a radical prostatectomy specimen has long been considered inherently adverse. Our study data suggest otherwise and indicate that high percentage tumor volume is associated with aggressive prostate cancer and consequently with a higher risk of multifocal positive surgical margins.

## 1 Introduction

Historically, patients and their physicians have feared pathological findings indicating a positive surgical margin (PSM) following radical excision of cancerous organs such as the bladder, pancreas, and prostate [1]. The immediate reaction is that the primary objective of complete removal of the tumor failed and the hope of cure is gone. However, our clinical understanding of PSMs and their impact on mortality following radical prostatectomy (RP) for prostate cancer (PC) dramatically changed over the past few years. As recently as 2005, PSMs were described as an established risk factor for biochemical recurrence (BCR) and assumed treatment failure [2]. Some have focused on the location of PSMs, but a consistent association is yet to be established [3]. Others have described varying BCR rates depending on the PSM characteristics, with margins >3 mm and unfavorable PSM pathology conferring a higher risk of BCR [4], [5], [6], [7]. However, Pellegrino et al [8] reported that in a cohort of 8141 men, only multiple PSMs (MPSMs) were associated with PC-specific mortality (PCSM). Of note, negative surgical margins (NSMs), unifocal PSMs (UPSMs) of <3 mm, and UPSMs of ≥3 mm had no impact on PCSM when compared to MPSM.

It is well known that more men will die with PC than from it. Average survival following RP is more than 25 yr [9] and hence quality of life (QOL) outcomes such as continence and sexual function are important [10], [11], [12], [13]. It has been demonstrated that greater membranous urethral length is associated with shorter time to continence recovery and better overall continence; however, the prevalence of margin positivity was greater for apical margins [14], [15].

The oncological landscape for PC continues to evolve and warrants further research and new perspectives. In PC, Gleason grade group (GG) largely drives long-term mortality outcomes, alongside pathological tumor stage [16]. Another factor that may play a role is the volume of disease [17], [18], [19], [20]. Interestingly, the question remains as to why the impact on PCSM does not vary between NSMs, UPSMs of <3 mm, and UPSMs of >3 mm, but is significantly associated with MPSMs. Logically, greater volume of disease should increase the chances of MPSMs. We hypothesized that higher pathological percentage tumor volume (PTV), rather than MPSMs, may increase the risk of PCSM because of more aggressive underlying disease and greater volume.

## 2 Patients and methods

Patients were prospectively entered into an anonymized, electronic database approved by the institutional review board at the University of California-Irvine (HS#1998-84). All data collection was conducted in compliance with the Health Insurance Portability and Accountability Act, and federal guidelines for informed consent were followed. We performed a retrospective cohort analysis of patients who underwent robot-assisted RP (*n* = 1552) for primary treatment of localized PC by a single surgeon from 2002 to 2018.

Preoperative demographics, oncological characteristics, and long-term follow-up data were collected for analysis. An extensive review of all pathology reports was conducted to identify margin characteristics and PTV. In general, all pathological reviews were performed under the guidance of trained genitourinary pathologists. Patients were excluded if they had missing information (*n* = 94), had undergone simple prostatectomy (*n* = 9), or had neuroendocrine/small-cell carcinoma (*n* = 3). Margin status was categorized as NSM, UPSM, or MPSM. PTV was categorized as 1–39% versus 40–100% for the primary comparison after evaluating other cut-points (eg, 20:80, 40:60).

The primary outcome was stepwise multivariate regression analysis for predictors of PCSM. The secondary outcome was the risk of 15-yr PCSM stratified by margin status and PTV via Kaplan-Meier analysis. Demographics were compared between groups using Student *t* test for continuous variables and a χ^2^ test for categorical variables. pT stage (≥pT3 vs pT2) and pathological GG (3–5 vs 1–2) were transformed into dichotomous variables for regression analyses. SPSS version 29 (IBM, Armonk, NY, USA) was used for all statistical analyses.

## 3 Results

Table 1 lists the baseline demographics stratified by margin status. In the study cohort, 1339 men had NSMs (86.3%), 172 had UPSMs (11.1%), and 41 had MPSMs (2.6%). Mean patient age varied significantly by margin status: NSM, 61.7 yr; UPSM, 63.9 yr; and MPSM, 65.3 yr (*p* = 0.008). Preoperative PSA also significantly differed by margin status: NSM, 6.66 ng/ml; UPSM 10.2 ng/ml; and MPSM, 12.2 ng/ml (*p* < 0.001). Margin status was also significantly associated with more advanced disease in terms of pathological GG, pT stage, and PTV, and long-term outcomes including BCR, PCSM, and overall mortality at 15 yr (Table 1).

**Table 1:** Patient demographics stratified by PSM focality

	NSM (*n* = 1339)	Unifocal PSM (*n* = 172)	Multifocal PSM (*n* = 41)	*p* value
Mean age, yr (SD)	61.7 (7.47)	63.9 (7.37)	65.3 (6.57)	0.008
Mean pPSA, ng/ml (SD)	6.66 (4.73)	10.2 (18.12)	12.2 (10.10)	<0.001
Gleason grade group, % (*n*)				<0.001
1	29.2 (391)	11.6 (20)	0.0 (0)	
2	42.3 (567)	39.5 (68)	26.8 (11)	
3	18.0 (241)	23.3 (40)	31.7 (13)	
4	3.7 (49)	4.7 (8)	0.0 (0)	
5	6.8 (91)	20.9 (36)	41.5 (17)	
pT stage, % (*n*)				<0.001
pT2	75.8 (1015)	30.2 (52)	12.2 (5)	
pT3	24.2 (324)	69.8 (120)	87.8 (36)	
PTV, % (*n*)				<0.001
1–19%	66.3 (887)	37.2 (64)	17.1 (7)	
20–100%	33.7 (452)	62.8 (108)	82.9 (34)	
PTV, % (*n*)				<0.001
1–39%	92.9 (1244)	76.7 (132)	43.9 (18)	
40–100%	7.1 (95)	23.3 (40)	56.1 (23)	
15-yr outcomes, % (*n*)				
Biochemical recurrence	17.0 (228)	44.2 (76)	68.3 (28)	<0.001
Overall mortality	5.5 (73)	11.6 (20)	14.6 (6)	<0.001
Prostate cancer mortality	0.7 (10)	3.5 (6)	9.8 (4)	<0.001

NSM = negative surgical margin; pPSA = preoperative prostate-specific antigen; PSM = positive surgical margin; PTV = percentage tumor volume; SD = standard deviation.

Initially, we compared demographics for groups stratified by PTV using cut-points of 20% versus 80%, and 40% versus 60% for optimal separation. Analysis revealed that a PTV cutoff of 40% versus 60% was more suitable than 20% versus 80% (Supplementary Fig. 1). Optimal separation was observed at PTV >40% (NSM 7.1%, UPSM 23.3%, and MPSM 56.1%). Table 2 lists demographic data for groups stratified at a PTV cutoff of 40%: 1394 patients had PTV of 1–39% and 158 had PTV of 40–100%. The 40–100% PTV group was significantly older (*p* = 0.017) and had higher preoperative PSA, pathological GG, and pT stage (*p* < 0.001). There were also significant differences in 15-yr rates for BCR, (17.6% vs 55.1%; *p* < 0.001), PCSM (5.4% vs 15.2%; *p* < 0.001), and OM (5.4% vs 15.2%; *p* < 0.001) for the 1–39% versus 40–100% PTV groups.

**Table 2:** Patient demographics stratified by PTV

	PTV 1–39% (*n* = 1394)	PTV 40–100% (*n* = 158)	*p* value
Mean age, yr (SD)	61.9 (7.50)	63.4 (7.40)	0.017
Mean preoperative PSA, ng/ml (SD)	6.67 (7.24)	11.8 (11.28)	<0.001
Gleason grade group, % (*n*)			<0.001
1	28.8 (402)	5.7 (9)	
2	43.0 (599)	29.7 (47)	
3	18.3 (255)	24.7 (39)	
4	3.4 (47)	6.3 (10)	
5	6.5 (91)	33.5 (53)	
pT stage, % (*n*)			<0.001
pT2	73.8 (1029)	27.2 (43)	
pT3	26.2 (365)	72.8 (115)	
Surgical margin status, % (*n*)			<0.001
Negative surgical margin	89.2 (1244)	60.1 (95)	
Unifocal positive margin	9.5 (132)	25.3 (40)	
Multifocal positive margin	1.3 (18)	14.6 (23)	
15-year outcomes % (*n*)			
Biochemical recurrence	17.6 (245)	55.1 (87)	<0.001
Overall mortality	5.5 (77)	15.2 (24)	<0.001
Prostate cancer mortality	0.6 (9)	7.0 (11)	<0.001

PSA = prostate-specific antigen; PTV = percentage tumor volume.

We performed stepwise regression analysis to identify significant predictors of PCSM. In the initial regression analysis, pT3 stage (odds ratio [OR] 10.27), pathological GG 3–5 (OR 6.67), and MPSMs (OR 5.29; *p* = 0.003) were significant predictors of PCSM (Table 3). However, when PTV was included in the regression analysis, the results showed that PTV was a significant predictor of PCSM (OR 3.63), but MPSMs were not (*p* = 0.105). UPSMs were not a significant predictor in either analysis.

**Table 3:** Stepwise multivariate regression results without and with PTV for prostate cancer–specific mortality

Parameter	OR (95% CI)	*p* value
**Without PTV**		
Age	0.976 (0.922–1.032)	0.395
Preoperative prostate-specific antigen	0.992 (0.952–1.033)	0.694
Stage pT3 (vs pT2)	10.269 (2.214–47.641)	0.003
Pathological Gleason grade group 3–5 (vs 1–2)	6.665 (1.874–23.711)	0.003
Unifocal positive margin (vs negative margin)	1.948 (0.729–5.210	0.184
Multifocal positive margin (vs negative margin)	5.287 (1.768–15.812	0.003
**With PTV**		
Age	0.990 (0.932–1.053)	0.760
Preoperative prostate-specific antigen	0.972 (0.914–1.034)	0.368
Stage pT3 (vs pT2)	16.393 (1.986–135.297)	0.009
Pathological Gleason grade group 3–5 (vs 1–2)	4.048 (1.107–14.797)	0.035
PTV 40–100% (vs 1–39%)	3.632 (1.338–9.860)	0.011
Unifocal positive margin (vs negative margin)	1.403 (0.470–4.186)	0.544
Multifocal positive margin (vs negative margin)	2.826 (0.804–9.931)	0.105

CI = confidence interval; OR = odds ratio; PTV = percentage tumor volume.

**Fig. 1:**
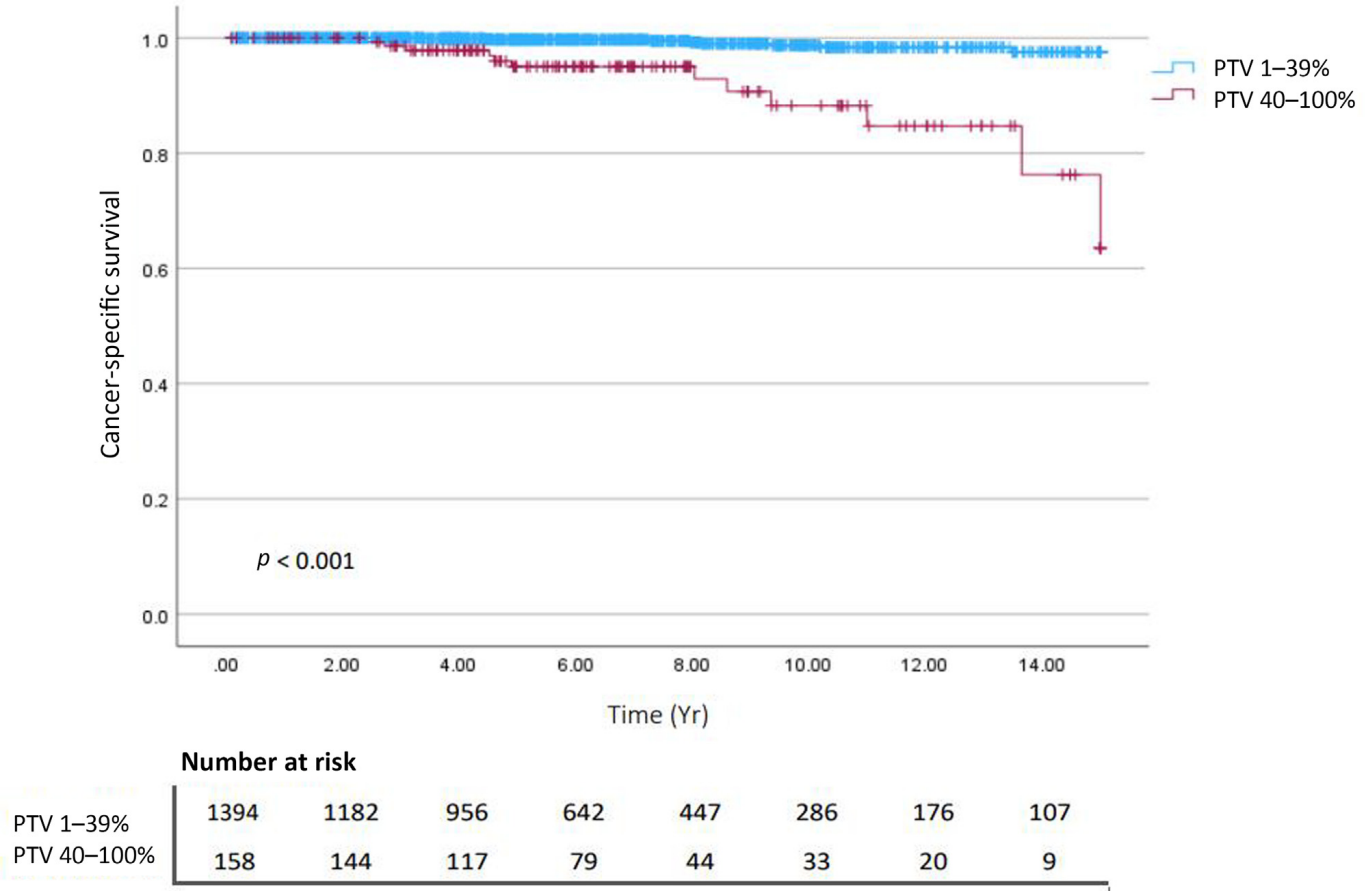
Kaplan-Meier curves for prostate cancer–specific survival stratified by percentage tumor volume (PTV).

**Fig. 2:**
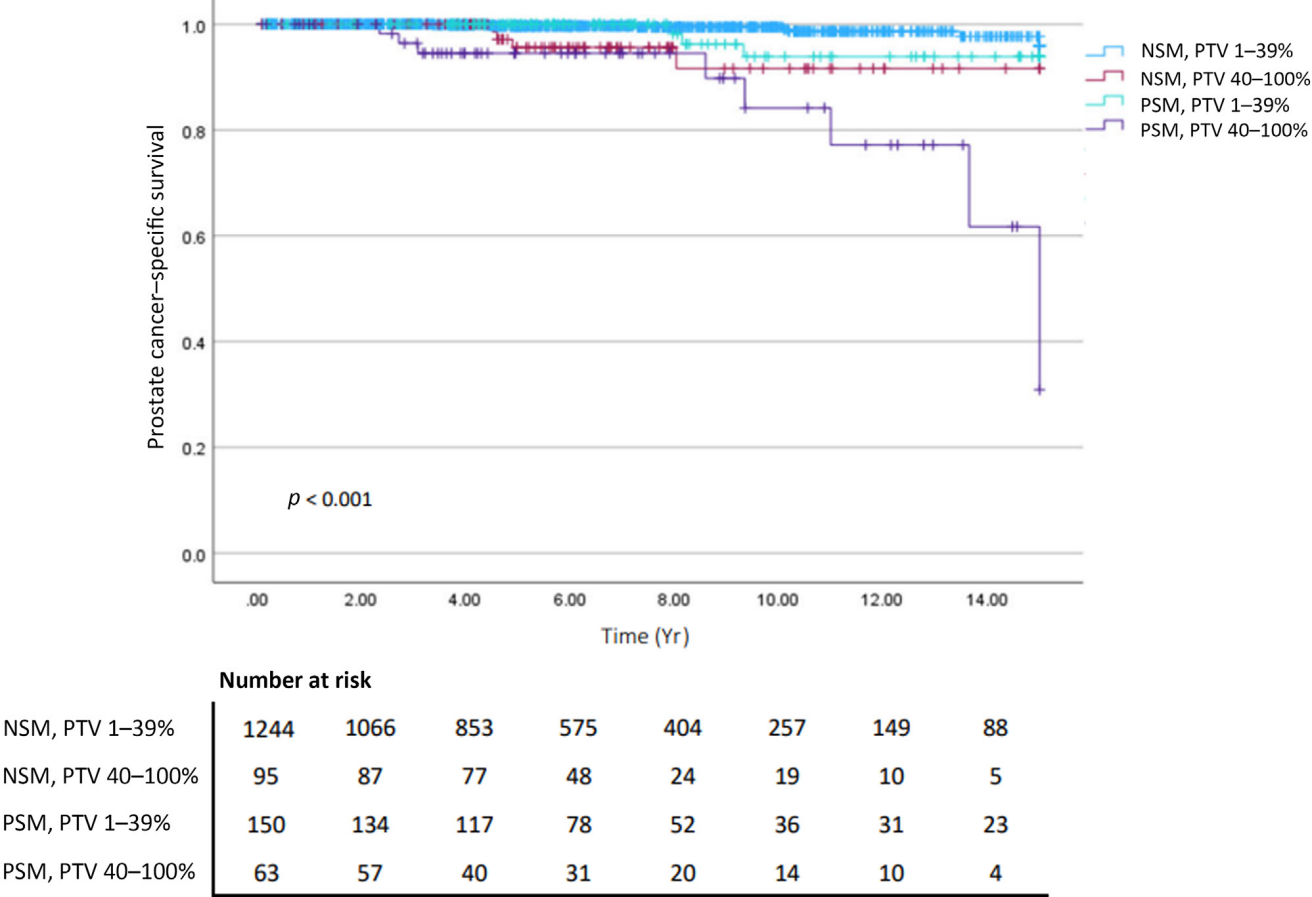
Kaplan-Meier curves for prostate cancer–specific survival stratified by margin status and percentage tumor volume (PTV). NSM = negative surgical margin; PSM = positive surgical margin.

Kaplan-Meier analysis of 15-yr outcomes revealed that 40–100% PTV was significantly associated with PCSM (*p* < 0.001; Fig. 1). Further stratification by PTV and margin status showed that only the combination of PSMs and 40–100% PTV was significantly associated with PCSM (*p* < 0.001; Fig. 2). Little difference was observed for the combination of PSMs and PTV 1–39% versus NSMs combined with either high or low PTV. An ad hoc analysis stratified by pathological GG and PTV revealed that only high grade (GG 3–5) and high PTV (40–100%) were associated with PCSM (*p* < 0.001; Supplementary Fig. 2). Subsequent patient stratification by pT stage and PTV revealed that only pT3 and high PTV were associated with PCSM (*p* < 0.001; Supplementary Fig. 3).

## 4 Discussion

A central question that emerges is whether surgeons should strive for NSMs in efforts to improve long-term oncological outcomes at the expense of sexual and urinary quality of life (QOL). The question is particularly pertinent when considering the potential consequences of wider resection, including deterioration in urinary and sexual functions [21], [22], [23]. While it is established that surgical technique is an important factor in PSM occurrence [24], the pathological finding of PSM is under scrutiny and, in contrast to the common perception, may not represent treatment failure, especially when weighed against QOL outcomes.

PSMs have long been identified as an adverse characteristic of robot-assisted RP outcomes [25], [26]. However, while there is an association between PSMs and BCR, the prognostic value of PSMs for PCSM remains a subject of ongoing debate [8], [25], [27], [28]. The primary goal of “radical” surgical procedures is to remove all the cancer, which is typically assumed to involve the organ in its entirety, without PSMs [29], [30]. Although this concept is nearly ubiquitously accepted for most cancer types, there is growing evidence that PC may be remarkably different, for which MPSMs have emerged as an adverse characteristic associated with PCSM [8]. There are no current explanations as to why MPSMs and not UPSMs predict more aggressive cancer. A possible explanation could be that an increase in PTV leads to an increase in aggressiveness and MPSMs. Ma et al [31] recently found that tumor volume on magnetic resonance imaging was predictive of PSMs for men undergoing nerve-sparing RP. Other studies have found that patients with higher PTV have higher PSM rates [27], [32].

Our stepwise multivariate analyses demonstrated that high PTV rather than MPSMs predicts PCSM. This is consistent with previous studies that found PTV or tumor volume calculated from prostate-specific membrane antigen–based imaging predicts adverse oncological outcomes in terms of PCSM [33], [34], [17], [18], [19]. Similar to the results reported by Pellegrino and colleagues [8], and in contrast to data for other cancers, we found that neither UPSMs nor MPSMs per se are inherently oncologically adverse. Rather, high PTV predicts highly aggressive PC and MPSMs.

A natural question then is whether knowing PTV preoperatively helps in guiding the surgical procedure. We performed sensitivity and specificity analyses for preoperative and postoperative pathology specimens. PTV demonstrated the highest sensitivity at 0.82, a positive predictive value of 0.93, and accuracy of 79.3%. We concluded that biopsy tumor volume is a practical clinical tool, whereas PTV offers significantly relevant data for hard metrics such as BCR and OM. Our findings further suggest that rather than wider resection to improve surgical margins, earlier diagnosis appears to be the best approach for reducing PTV and disease severity and improving PCSM. Hence, early and very cautious postoperative monitoring of PSA for aggressive intervention should be based on PTV, along with pT stage and pathological GG findings.

Limitations of our study include the retrospective review of prospective data and small numbers of cases with MPSM and high PTV.

## 5 Conclusions

Our results provide compelling evidence that MPSMs, like unifocal PSMs, are not inherently associated with adverse PCSM outcomes. Rather, MPSMs signify more aggressive underlying disease that is responsible for adverse oncological outcomes. The data emphasize the importance of PTV, pathological GG, and pT stage as predictors of PCSM. In contrast to conventional prioritization, this study adds to growing evidence that PSMs, including MPSMs, alone do not warrant surgical interventions that increase the risk of urinary and sexual function complications in the name of reduced PCSM.

  ***Author contributions:*** Thomas Ahlering had full access to all the data in the study and takes responsibility for the integrity of the data and the accuracy of the data analysis.

  *Study concept and design*: Ahlering, Tran, Zeidan.

*Acquisition of data*: Zeidan, Nguyen, Hwang, Zhang.

*Analysis and interpretation of data*: Ahlering, Tran, Hwang.

*Drafting of the manuscript*: Zeidan, Tran, Hwang, Ahlering.

*Critical revision of the manuscript for important intellectual content*: Ahlering, Huynh.

*Statistical analysis*: Zeidan, Tran.

*Obtaining funding*: None.

*Administrative, technical, or material support*: Huang.

*Supervision*: Ahlering.

*Other*: None.

  ***Financial disclosures:*** Thomas Ahlering certifies that all conflicts of interest, including specific financial interests and relationships and affiliations relevant to the subject matter or materials discussed in the manuscript (eg, employment/affiliation, grants or funding, consultancies, honoraria, stock ownership or options, expert testimony, royalties, or patents filed, received, or pending), are the following: None.

  ***Funding/Support and role of the sponsor:*** None.

  ***Data sharing statement:*** The data presented in this study are available on request from the corresponding author.
