# Supplementary material for: Impact of Surgical Margin Status and Tumor Volume on Mortality After Robotic Radical Prostatectomy
Source: Eur Urol Open Sci. 2024 Dec 31;71:187–92. doi: 10.1016/j.euros.2024.12.004 (PMC11751535; doi:10.1016/j.euros.2024.12.004)

**Supplementary material**

Supplementary Fig 1. Confusion matrix on cutoffs between 40:60 and 20:80 PTV

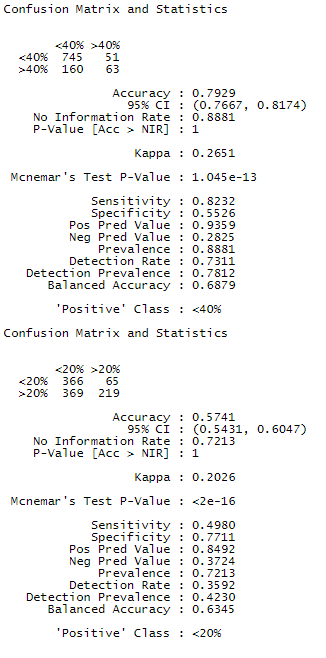


**Supplementary Table 1.** Univariate Regression Analysis for PCSM

|  | **OR** | **95% CI** | **Sig.** |
| --- | --- | --- | --- |
| Age | 1.027 | 0.973 - 1.083 | 0.335 |
| Pre-op PSA | 1.015 | 0.996 - 1.035 | 0.132 |
| pStage 3 (vs. 2) | 26.786 | 6.291 - 114.056 | <0.001 |
| pGGG 3-5 (vs. 1-2) | 16.162 | 4.815 - 54.246 | <0.001 |
| PTV 40-100% (vs. 1-39%) | 12.658 | 5.245 - 30.544 | <0.001 |
| PSM-Unifocal (vs NSM) | 4.630 | 1.799 - 11.915 | 0.001 |
| PSM-Multifocal (vs NSM) | 19.514 | 6.937 - 54.869 | <0.001 |

Supplementary Figure 2 –


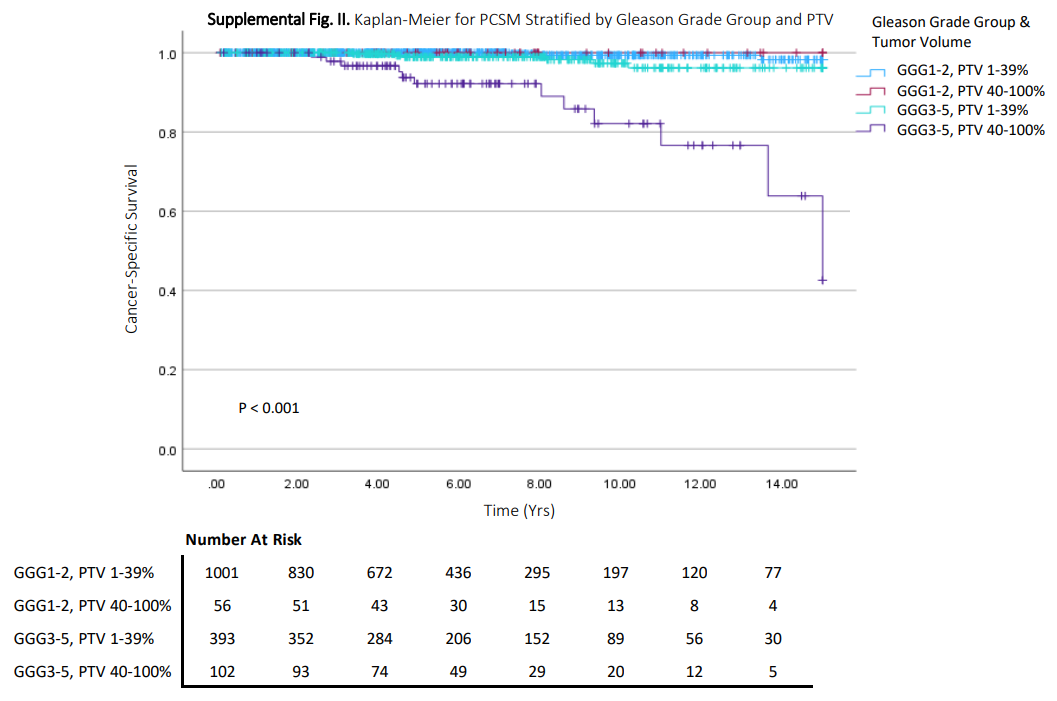


Supplementary Figure 3


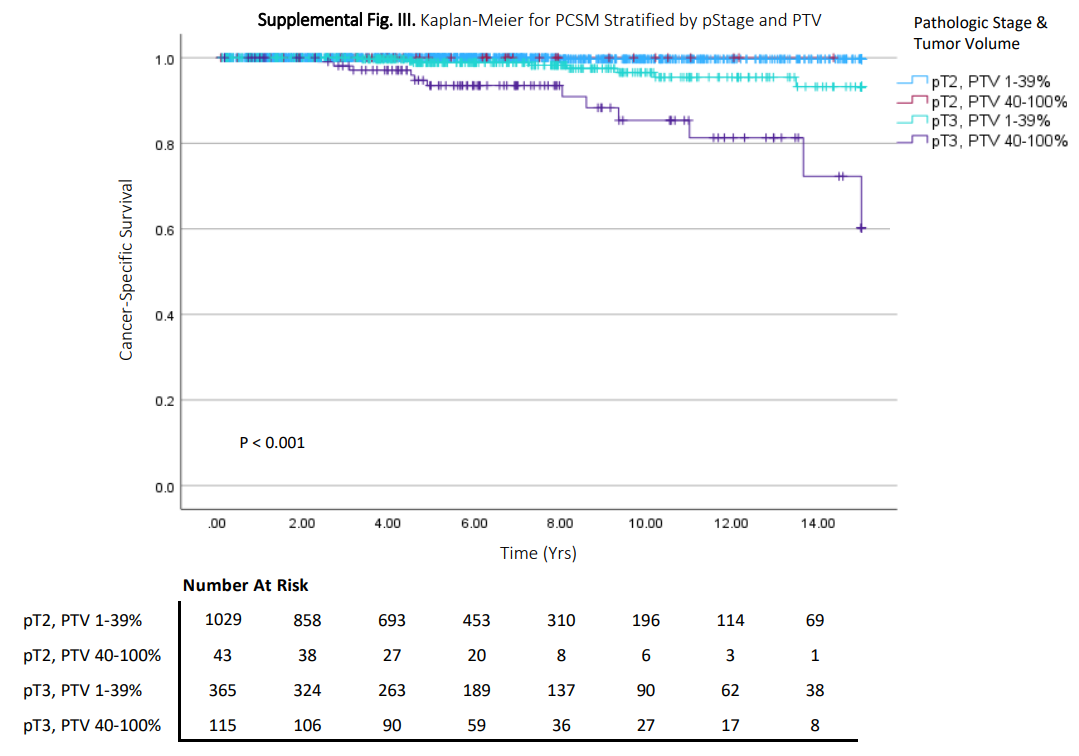

Supplement: Supplementary Data 1 [file mmc1.docx]
